# A Multi-Purpose Approach to the Mechanisms of Action of Two Biocides (Benzalkonium Chloride and Dibromonitrilopropionamide): Discussion of *Pseudomonas fluorescens*’ Viability and Death

**DOI:** 10.3389/fmicb.2022.842414

**Published:** 2022-02-18

**Authors:** Ana C. Barros, Luis F. Melo, Ana Pereira

**Affiliations:** ^1^LEPABE-Laboratory for Process Engineering, Environment, Biotechnology and Energy, Department of Chemical Engineering, Faculty of Engineering, University of Porto, Porto, Portugal; ^2^ALiCE - Associate Laboratory in Chemical Engineering, Faculty of Engineering, University of Porto, Porto, Portugal

**Keywords:** benzalkonium chloride, dibromonitrilopropionamide, mechanism of action, viability, dead cells

## Abstract

Biocides are widely used in water treatment for microbiological control. The rise of antimicrobial resistance and the need to assure properly managed water systems require a better understanding of the mechanisms of action of biocides and of their impact on cell’s viability as a function of dosage concentrations. The present work addresses these two aspects regarding the biocides benzalkonium chloride (BAC) and dibromonitrilopropionamide (DBNPA)—two biocides commonly found in the water treatment industry. For that, the following parameters were studied: culturability, membrane integrity, metabolic activity, cellular energy, and the structure and morphology of cells. Also, to assess cell’s death, a reliable positive control, consisting of cells killed by autoclave (dead cells), was introduced. The results confirmed that BAC is a lytic biocide and DBNPA a moderate electrophilic one. Furthermore, the comparison between cells exposed to the biocides’ minimum bactericidal concentrations (MBCs) and autoclaved cells revealed that other viability parameters should be taken into consideration as “death indicators.” The present work also shows that only for the concentrations above the MBC the viability indicators reached values statistically similar to the ones observed for the autoclaved cells (considered to be definitively dead). Finally, the importance of considering the biocide mechanism of action in the definition of the viability parameter to use in the viable but non-culturable (VBNC) determination is discussed.

## Introduction

For decades, biocides have been used in a wide range of applications, such as: water treatment, domestic environment, food preservation, healthcare sanitation, textile, and other industries to control and kill bacteria (Kahrilas et al., 2015; Jones and Joshi, 2021). Biocides are defined as substances with one or more active ingredients that are used to inactivate, prevent, or control any harmful microorganisms by chemical or biological ways (Ribeiro et al., 2018).

The same biocide can be used for several applications, sometimes by changing only the employed concentration. Additionally, biocides can have both bacteriostatic and bactericidal effects, which generally relies on the applied concentration (Denyer, 1990).

Although the final result can be the same (cell death), the mechanisms by which bacteria reach that state can be completely different. Many mechanisms of antimicrobial action were described and most times the antimicrobial action of a biocide is a combination of several mechanisms (Liwa and Jaka, 2015). What distinguishes the different mechanisms of action is the target site where they act. So, biocides can: (i) interfere with the replication of genetic information (nucleic acids); (ii) interfere with protein synthesis; (iii) alter structure and function of cell wall; (iv) increase permeability and disrupt the cytoplasmatic membrane; and (v) inhibit intermediate metabolic pathways (Liwa and Jaka, 2015).

In short, biocides can be classified in two main categories: electrophilic and membrane active biocides (Akhova and Tkachenko, 2014).

Electrophilic biocides include the extreme electrophilic (oxidants) and the moderate electrophilic (which includes most of the non-oxidizing biocides; Amjad, 2010; Bajpai, 2015). The oxidants, as the name suggests, oxidize organic material by either releasing toxic free radicals or by halogenating molecules within a cell, and have a rapid killing rate (Chapman, 2003; Amjad, 2010; Bajpai, 2015). The moderate electrophilic biocides interact with cellular nucleophiles by covalent linkage, which causes enzyme inactivation. Isothiazolones, dibromonitrilopropionamide (DBNPA), glutaraldehyde, formaldehyde, bronopol, and inorganic metallic ions (mercury, silver, and copper) fall in this category (Chapman, 2003; Paulus, 2005; Amjad, 2010; Bajpai, 2015).

The membrane active category includes the lytic biocides and the protonophores (Amjad, 2010; Bajpai, 2015). Lytic biocides, like quaternary ammonium compounds (QACs), alcohols, and biguanides, lead to cell lysis. They first interact with bacterial outer membrane and then with the cytoplasmatic membrane, which causes loss of integrity and leakage of intracellular components, followed by cell lysis (Paulus, 2005; Amjad, 2010). On the other hand, the protonophores includes weak acids, such as citric acid, benzoic acid, and sorbic acid. These biocides interfere with the pH balance of cells, which can cause cytoplasm acidification and consequent disruption of the proton-motive force. Thereby, the flow of protons across the cytoplasmatic membrane is compromised, meaning that cells cannot produce more energy (adenosine triphosphate, ATP) and that once the ATP sources end up, the cell has no more energy for its basic metabolism and starts to die (McDonnell, 2007; Amjad, 2010).

The current and wide spread use of disinfectant products containing low doses of biocides has raised some attention, as it can lead to the development of microbial tolerance to biocides and to the regrowth of microorganisms due to the inefficacy of the biocidal treatment (Maillard, 2005; Wesgate et al., 2016). The knowledge of the mechanisms of action of biocides is primordial to establish more accurate and rational disinfection strategies to combine different biocides to reinforce the antimicrobial activity and avoid potential tolerance mechanisms and toxicological effects.

The present work focuses on two commonly used biocides for industrial water treatment purposes [Benzalkonium chloride (BAC) and DBNPA], which have distinct modes of action. Benzalkonium chloride belongs to the family of QACs, meaning that they have a positively-charged quaternary amine group and one or two alkyl chains (Pati and Arnold, 2020). QACs are lytic cationic biocides which act through the interaction of their positively-charged part with the negatively-charged phospholipid bilayer of the bacterial cell membrane (Pati and Arnold, 2020). Additionally, membrane destabilization prompts cell lyses and intracellular components start to leak, leading to cell death (McDonnell, 2007; Gerba, 2015).

Dibromonitrilopropionamide is a moderate electrophilic biocide that interacts directly with cellular components (Siddiqui et al., 2017; Kucera, 2019). This biocide enters the cell by diffusion, thereby it takes a little longer to kill (Kucera, 2019). Its mechanism of action relies on the interaction with thiol groups of proteins (e.g., cysteine or glutathione). This reaction leads to the formation of disulphide bonds and the consequent irreversible protein damage. It also interferes with the transport across the cell and stops respiration and ATP synthesis (Ullah, 2011; Siddiqui et al., 2017; Kucera, 2019).

Although a significant number of authors have proposed hypotheses for the mechanism of action of DBNPA (Paulus, 2005; Williams and McGinley, 2010; Campa et al., 2019), there is a lack of studies based on a consistent set of assays to prove such hypotheses. So, the present paper aims to provide insights into the reported mechanisms of action of DBNPA. For that, *Pseudomonas fluorescens* cultures were exposed to different biocide concentrations, and several parameters evaluated, including: culturability, membrane integrity, metabolic activity, cellular energy, and morphological/structural changes. For comparison purposes, BAC was equally tested since its mechanism of action has been widely described in the literature (Chapman, 2003; Amjad, 2010; Bajpai, 2015). Furthermore, the antimicrobial activity of the biocides is here discussed against a negative control of live cells and a positive control of dead cells (by autoclaving). This strengthens the discussion by providing insights on the extent of the cell “damage” toward its dead state.

## Materials and Methods

### Biocides

Benzalkonium chloride was purchased from Sigma-Aldrich. The biocide 2,2-Dibromo-3-nitrilopropionamide (DBNPA), 10% (w/w) of active ingredient was provided by Enkrott®, S.A. Stock solutions of the selected biocides were aseptically prepared in ultrapure water (UPW) and stored at 4°C.

### Microorganisms and Culturing Conditions

*Pseudomonas fluorescens* was isolated from a drinking water distribution system and identified by 16S rRNA gene sequencing as described by Ferreira et al. (2009).

Bacterial cells were grown overnight in a batch culture in a nutrient medium with the following composition (per liter): 5 g glucose (Fisher Chemical), 2.5 g peptone (Oxoid), and 1.25 g yeast extract (Oxoid), in 0.02 M phosphate buffer pH 7 (KH2PO4; Na2HPO4) at 30°C and under agitation (160 rpm).

### Minimum Bactericidal Concentration Determination

An overnight growth culture was harvested by centrifugation at 4,000 rpm for 10 min and washed twice with sterile saline solution at 0.85% (v/v). Then, bacteria were resuspended in saline solution and the optical density (at 610 nm) adjusted to 0.2 ± 0.02 (1.3 × 10^8^ CFU/mL). The antimicrobial activity of biocides against planktonic bacteria was performed according to the European Standard EN 1276 (European Committee Standardisation, 2010) with some modifications. Bacterial cells (100 mL) were placed in 250-mL flasks under agitation (160 rpm) at 25°C. For each biocide, different concentrations were tested (see section “Biocide Exposure”). The exposure times tested were: 0, 0.17, 0.5, 1, 5, 15, 30, and 60 min. At each time point an aliquot of 100 μl was collected and subjected to a biocide neutralization step (10 min contact time) using a universal neutralizer: 3 g/L of lecithin (Alfa Aesar), 30 g/L of saponin (VWR Chemicals), 1 g/L of L-histidine (Merck), 5 g/L of sodium thiosulphate (Labkem), and 30 g/L of polysorbate 80 (VWR Chemicals) in 0.0025 M phosphate buffer. Afterward, the appropriate dilutions were performed and the cells plated on PCA plates and incubated at 30°C. After 24 h, the colony forming units (CFU) were enumerated. The minimum bactericidal concentration (MBC) was determined as the lower concentration able to kill 99.9% of bacteria (Parvekar et al., 2020) within 30 min. Three independent experiments were performed for each condition.

### Mechanisms of Action of the Biocides

#### Biocide Exposure

Bacterial cells from an overnight culture were centrifuged (4,000 rpm, 10 min) and washed with saline solution. Afterward, the optical density (at 610 nm) was adjusted to 0.2 ± 0.02 in saline solution. Then, bacterial cells were placed in contact with the biocidal solutions for 30 min at 25°C and 160 rpm. Four different concentrations of each biocide were used, namely: the concentration determined as the MBC, one concentration above the MBC, and two concentrations below the MBC. Therefore, the selected concentrations were 30, 40, 100, and 160 mg/L for BAC and 5, 8, 10, and 35 mg/L for DBNPA. Controls were performed with both live cells only (negative control) and dead cells only (positive control). To obtain dead cells, bacterial cells were autoclaved at 121°C for 20 min. The autoclaving process led to microbial inactivation percentages of: ~100, 99, and 87% in terms of culturability, ATP and metabolic activity, respectively. Henceforth, the live cells suspension and the dead cells suspension will be called “C^−^” and “C^+^,” respectively.

After biocide exposure, the cells were neutralized as described in section “Minimum Bactericidal Concentration Determination,” centrifuged (4,000 rpm, 5 min), washed twice (to remove biocide) and then resuspended in saline for further analysis.

To fully understand the mechanisms of antimicrobial action of the selected biocides, the following parameters were evaluated after biocide exposure, namely: (i) culturability; (ii) membrane integrity; (iii) metabolic activity; (iv) cellular energy; and (v) structural and morphological changes.

#### Culturability

Bacterial cells were serially diluted in saline solution and plated on plate count agar (PCA) using the drop plate method (Reed and Reed, 1948). Then, the plates were incubated at 30°C for 24 h. Afterward, colonies were enumerated and the results expressed as logarithm of the colony forming units per milliliter (CFUs/mL). Three independent experiments were performed for each condition.

#### Membrane Integrity

The LIVE/DEAD® *Bac*light™ kit (Invitrogen) was used to assess membrane integrity of bacterial cells after exposure to biocides. In brief, an aliquot of 1 mL was filtered through a 0.2-μm Nucleopore® (Whatman) black polycarbonate membrane and stained with 250 μl of SYTO9™ and 50 μl of propidium iodide (PI) in the dark (Barros et al., 2021). After reacting for 7 min, the stains were filtered and the samples mounted on a slide with immersion oil. Then, samples were observed using a 100x objective oil immersion fluorescence objective of a LEICA DMLB2 microscope incorporated with a mercury lamp HBO/100W/3 and a CCD camera. The optical filters used were a combination of a 480–500 nm excitation filter and a 485 nm emission filter (Chroma 61000-V2 DAPI/FITC/TRITC; Ferreira et al., 2011). A IM50 software (LEICA) was used to record images. At least 15 images were captured for each sample and the experiments were repeated in three different occasions.

#### Metabolic Activity

Stock solutions of resazurin (400 μM) were prepared in sterile ultrapure water and stored at −20°C. In brief, 190 μl of samples and 10 μl of resazurin were added in the dark to each well. Then, the fluorescence was recorded at λexcitation = 570 nm and λemission = 590 nm every 30 min for 24 h using a FLUOstar® Omega microtiter plater reader (BMG LABTECH, Germany). Blank assay was performed using saline solution. The results were presented in terms of relative fluorescence units (RFU). Three independent experiments with triplicates were performed for each case.

#### Cellular Energy (ATP)

Adenosine triphosphate was measured using BacTiter-Glo™ Microbial Cell Viability Assay (Promega, Madison, Wisconsin, United States) according to the manufacturer’s instructions. Briefly, 100 μl of samples was mixed with 100 μl of BacTiter-Glo™ reagent in white 96-well plates and allowed to interact for 5 min at room temperature. Then, luminescence was recorded using the lens from a FLUOstar® Omega microtiter plater reader (BMG LABTECH, Germany) and the output values were recorded in relative light units (RLU). Background luminescence was removed by recording luminescence of control wells without cells. Three independent experiments with triplicates were performed for each case.

#### Transmission Electron Microscopy Analysis

For the ultrastructure analysis, cells were fixed in a solution of 2% glutaraldehyde (#16316; Electron Microscopy sciences) with 2.5% formaldehyde (#15713; Electron Microscopy sciences) in 0.1 M sodium cacodylate buffer (pH 7.4) for 24 h, at room temperature, and post fixed in 2% osmium tetroxide (#19190; Electron Microscopy Sciences) diluted in 0.1 M sodium cacodylate buffer. After centrifugation, the pellet was resuspended in Histogel™ (Thermo, HG-4000-012) and then stained with aqueous 1% uranyl acetated solution overnight, dehydrated and embedded in Embed-812 resin (#14120; Electron Microscopy sciences). Ultra-thin sections (50 nm thickness) were cut on a RMC Ultramicrotome (PowerTome, United States) using Diatome diamond knifes, mounted on mesh copper grids (Electron Microscopy Sciences), and stained with uranyl acetate substitute (#11000; Electron Microscopy Sciences) and lead citrate (#11300; Electron Microscopy Sciences) for 5 min each. Samples were viewed on a JEOL JEM 1400 transmission electron microscope (JEOL, Tokyo, Japan) and images were digitally recorded using a CCD digital camera Orius 1100W (Tokyo, Japan). The transmission electronic microscopy (TEM) was performed at the HEMS core facility at i3S, University of Porto, Portugal with the assistance of Ana Rita Malheiro and Rui Fernandes.

### Statistical Analysis

Statistical analysis was performed using Graphpad Prism version 9.0.1 for macOS software (GraphPad Software, San Diego, CA, United States). Data was analyzed using one-way ANOVA with Tukey’s multiple comparison test. A confidence level of 95% (*p* < 0.05) was used as statistical significance. All data was expressed as means ± SD of three independent experiments.

## Results and Discussion

### Minimum Bactericidal Concentration

**Figure 1:**
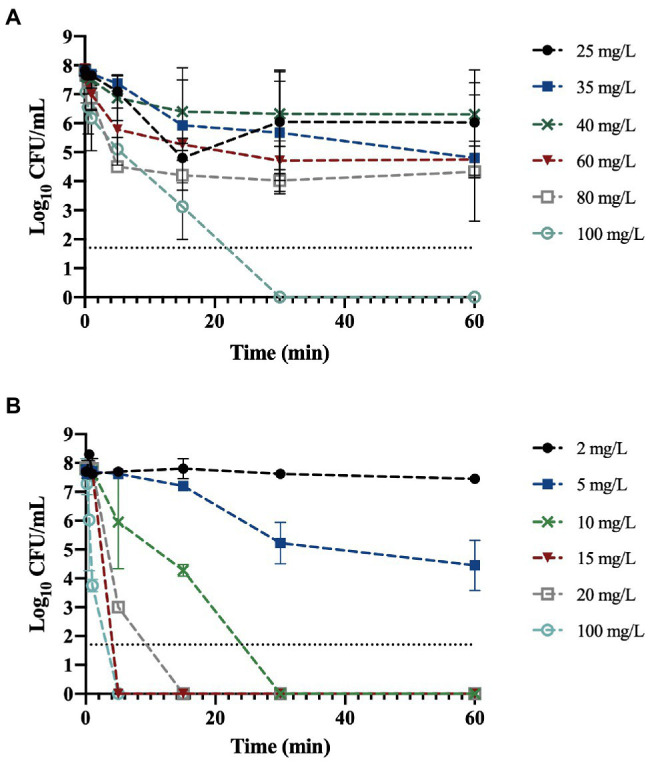
Logarithm of the colony forming unit per milliliter (CFU/mL) of planktonic *Pseudomonas fluorescens* exposed to: **(A)** benzalkonium chloride (BAC) and **(B)** dibromonitrilopropionamide (DBNPA) at different concentrations along time. The line indicates the method detection limit (1.70 log_10_ CFU/mL). The means ± SD of three independent experiments are illustrated.

The antimicrobial activity of BAC and DBNPA was tested and the resulting time-kill curves are presented in Figure 1. As can be observed from Figure 1, both biocides were able to completely inactivate bacterial cells in less than 1 h of contact time.

Figure 1A shows that the exposure of *P. fluorescens* to BAC at 25, 35, and 40 mg/L, did not affect the number of culturable cells (*p* > 0.05). On the other hand, at 60 min of contact time, a 1.3 and 1.8-log_10_ reduction was observed for 60 and 80 mg/L, respectively.

As antimicrobial activity of biocides is dose and time-dependent (Grobe et al., 2002; Fernandes et al., 2020) the MBC was determined. The MBC corresponds to the lowest concentration of biocide that is able to kill 99.9% of the initial bacterial cells (Parvekar et al., 2020). The only concentration able to reduce culturability to zero (6.3-log_10_ reduction) was 100 mg/L, so this concentration was assumed to be the MBC for BAC. Fazlara and Ekhtelat (2012) obtained an MBC of 80 mg/L for *Pseudomonas aeruginosa*, which is lower than the value obtained in the present study; although, their bacterial concentration was also lower (10^6^ CFU/mL). On the other hand, Ko et al. (2007) obtained a MBC of 16 mg/L for both *P. aeruginosa* and *Pseudomonas putida* in bold medium, which is six times lower than the value obtained in this study (although the initial bacterial load was not specified). Additionally, in the same study, the MBC determination in a different culture medium (Mueller-Hinton Broth) resulted in different MBC values, namely, 63 and 32 mg/L for both *P. aeruginosa* and *P. putida*, respectively.

Regarding Figure 1B, it is noticeable that DBNPA at 2 mg/L did not affect the culturability of bacterial cells, even after 1 h of contact time. The exposure of *P. fluorescens* cells to 5 mg/L of DBNPA resulted in a 0.3 and 1.3-log_10_ reduction (*p* < 0.05) after 30 and 60 min of contact time, respectively. Exposing the cells to 10 mg/L led to an 6.3-log_10_ reduction of bacterial culturability within 30 min. Therefore, the MBC of DBNPA was found to be 10 mg/L. The differences observed between the concentrations of either 2 or 5 mg/L and all the other concentrations were statistically significant (*p* < 0.05). Russo (2016) found that the MBC of DBNPA against *Escherichia coli* in Mueller-Hinton Broth after 24 h of exposure was 20 mg/L. In the study of Ko et al. (2007), the MBC of DBNPA was 128 and 8 mg/L for *P. putida* and *P. aeruginosa*, respectively.

The differences observed in the MBC values for both biocides show that the MBC is dependent on the microorganism species/strain, the culture medium, and the method used to evaluate it (Bridier et al., 2011).

### Mechanisms of Antimicrobial Action of Biocides

In order to understand the mechanisms of antimicrobial action of BAC and DBNPA, bacterial cells were exposed to biocides for 30 min, at four different biocidal concentrations. The choice of a 30 min contact time follows the discussion undertaken about the MBC and the minimum time to achieve non-culturability for both biocides. The biocidal concentrations were chosen to meet the MBC, two concentrations below the MBC and one concentration above it. After the exposure to the selected biocides several parameters were analyzed, namely: culturability, membrane integrity, metabolic activity, cell energy, and cell morphology. For interpretation and comparison purposes, these parameters were also assessed for the controls: negative control (C^−^) corresponding to live cells of *P. fluorescens* and the positive control (C^+^) from the autoclaved cells (dead cells).

#### Effect of Biocides on Culturability

**Figure 2:**
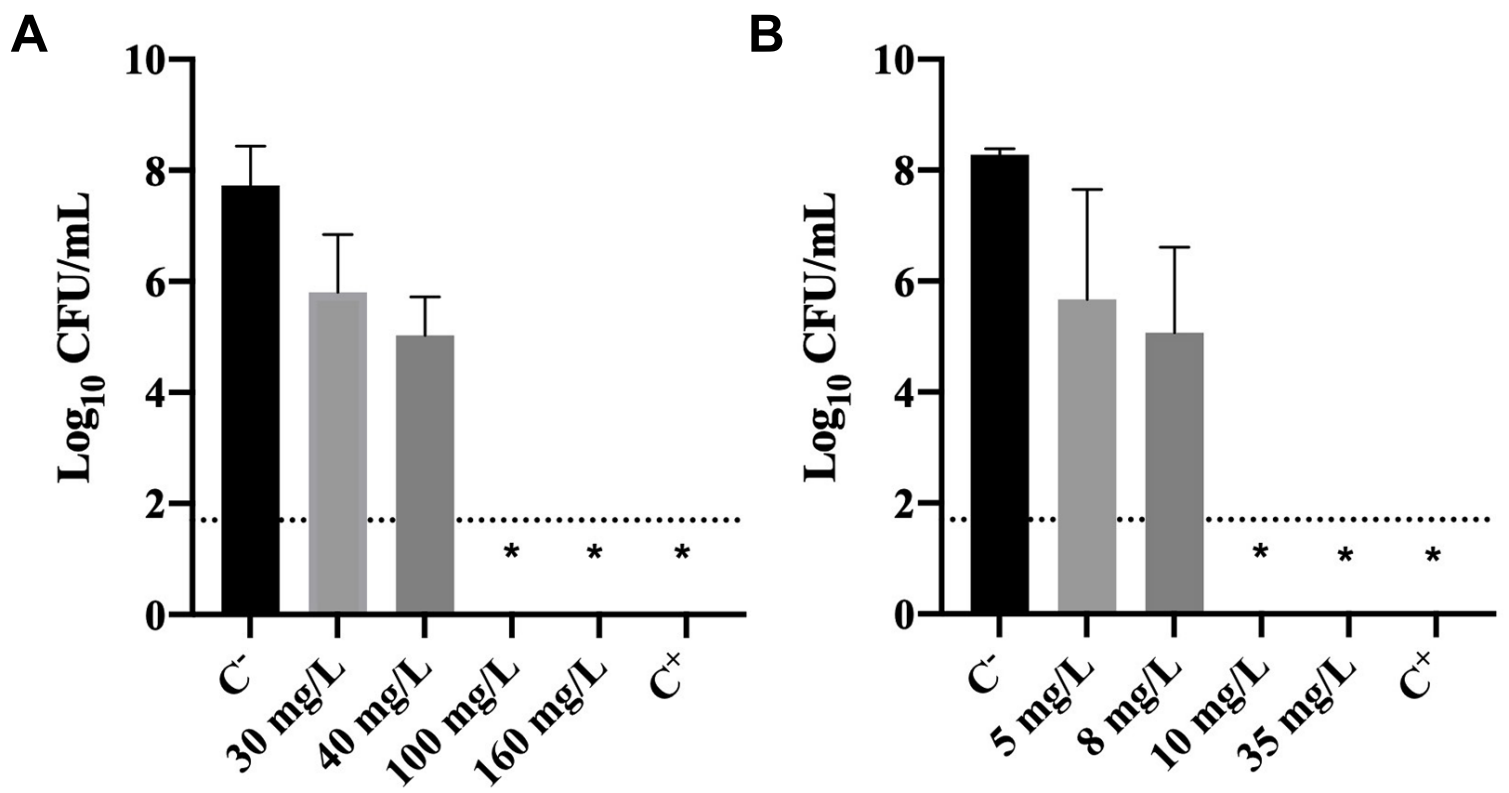
Logarithm of the CFU/mL of *Pseudomonas fluorescens* cells after being exposed for 30 min to different concentrations of **(A)** BAC and **(B)** DBNPA. Controls were performed with only live cells (C^−^) and dead cells, killed by autoclave (C^+^). The means ± SD of three independent experiments are presented. The line indicates the method detection limit (1.70 log_10_ CFU/mL). The symbol * means that no CFUs were detected.

The culturability of cells previously exposed to BAC and DBNPA was evaluated and the results are presented in Figure 2.

The results illustrated in Figure 2 show that total inactivation (no CFU counts) of bacterial cells was obtained for: (i) 100 and 160 mg/L of BAC; (ii) 10 and 35 mg/L of DBNPA; and (iii) autoclaved cells (C^+^). These results are not surprising as these concentrations correspond to the MBC’s and concentrations above the MBC (Figure 1) or, in the case of C^+^, to the positive control (dead cells).

Figure 2A also shows that the lowest BAC concentrations tested, 30 and 40 mg/L, affected the culturability of cells as they resulted in a 0.3 and 1.3 log_10_-reduction, respectively. Similarly, the lowest DBNPA concentrations (5 and 8 mg/L) also reduced the culturability in 0.2 and 1.3 log_10_, respectively.

#### Effect of Biocides on Membrane Integrity

The membrane integrity was evaluated using two different dyes: SYTO 9 and PI. PI is only able to penetrate cells with damaged membranes; it binds to nucleic acids and emits a red fluorescent light. SYTO9, which also binds to nucleic acids, is used as a viability marker as it enters all cells, regardless their membrane integrity, staining cells green (Ding et al., 2017).

**Figure 3:**
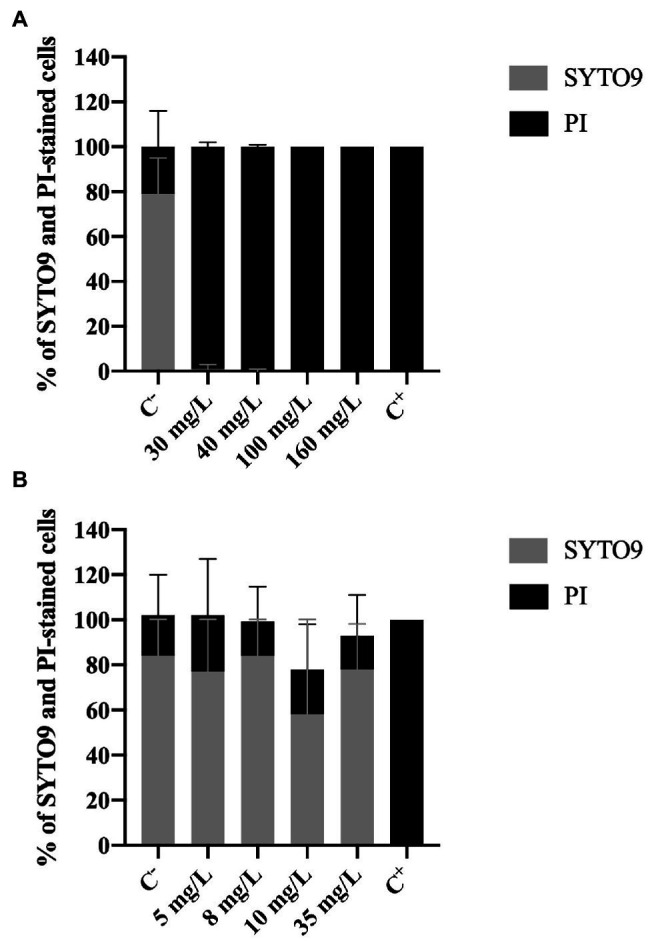
Percentage of *Pseudomonas fluorescens* cells stained with SYTO9 (Green cells) and propidium iodide (Red cells) after being exposed for 30 min to different concentrations of **(A)** BAC and **(B)** DBNPA. Controls were performed with only live cells (C^−^) and dead cells, killed by autoclave (C^+^). The means ± SD of three independent experiments are presented.

Figure 3A shows an increase of PI-stained cells with increasing BAC concentrations. The positive control presented 79 and 21% of SYTO9 and PI-stained cells, respectively. On the other hand, total loss of membrane integrity (100% PI uptake) was observed for the concentrations equal to or above 30 mg/L, as well as for the negative control (*p* < 0.0001). Fernandes et al. (2020) also studied the membrane integrity of *P. fluorescens* after being exposed to BAC (10 and 100 mg/L) and found similar values of PI uptake percentage (84–100%).

On the other hand, no statistical differences (*p* > 0.05) were observed in the PI and the SYTO9 uptake between C^−^ and DBNPA-exposed cells (Figure 3B). These PI uptake values (25, 15, 20, and 15%, respectively for 5, 8, 10, and 35 mg/L) are also similar to the ones observed in the negative control (C^−^). Since the C^−^ corresponds to live cells in the growth phase, these results suggest that the DBNPA did not have a significant impact on the membrane integrity of *P. fluorescens*. Additionally, and as expected, the autoclaved cells (C^+^) showed total loss of membrane integrity (100% PI uptake).

It is important to note that the PI uptake percentages observed for C^−^ (18–21%) are consistent with data reported in the literature (Fernandes et al., 2020; Barros et al., 2021). The question why live cells can be stained with PI remains unanswered (Shi et al., 2007). Some authors (Shi et al., 2007) showed that active growing cells can have, for short periods of time, their membrane’s integrity affected. Other studies (Kirchhoff and Cypionka, 2017) attributed the PI uptake by intact live cells to the existence of a high membrane potential.

In short, results from Live/Dead assay clearly demonstrate that the two tested biocides have a different impact at the cells membrane level: BAC disrupts cells membranes, while DBNPA does not affect the cells membrane integrity (independently of inactivating the cells, or not). The interpretation of these results will be further developed in sub-section “The Issue of Viable but Non-culturable Cells,” when addressing the issue of viable but not culturable cells.

#### Effect of Biocides on Metabolic Activity

Metabolic activity of cells was determined using the resazurin dye assay. Resazurin is commonly used to assess bacterial viability. Basically, resazurin is a weakly-fluorescent dye that is reduced to a pink highly-fluorescent dye (resorufin). This reduction only occurs in the presence of metabolically active cells (Bueno et al., 2002; Csepregi et al., 2018). Therefore, the intensity of fluorescence can be correlated to the presence of metabolically active cells (viable cells).

**Figure 4:**
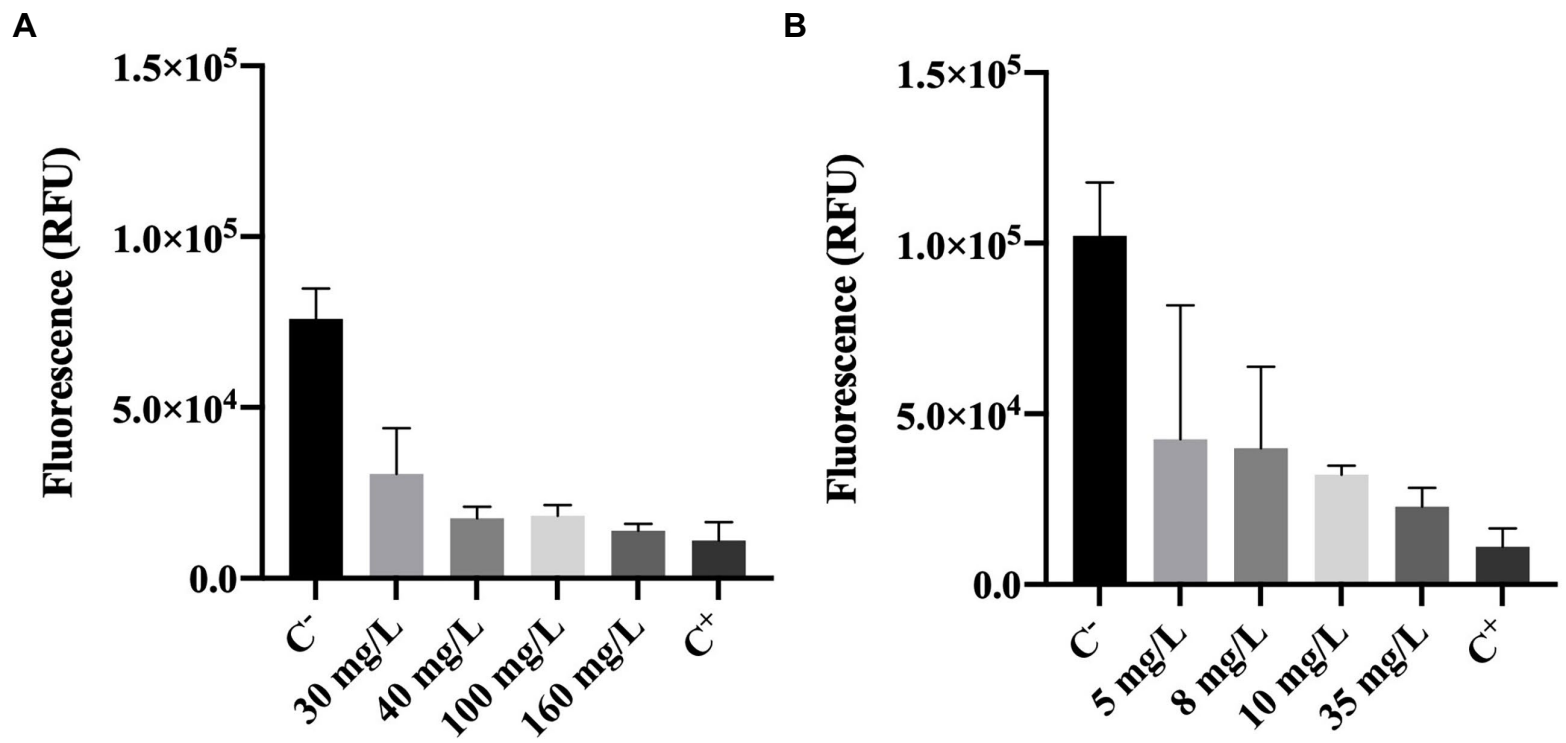
Fluorescence of *Pseudomonas fluorescens* cells stained with resazurin after being exposed for 30 min to different concentrations of **(A)** BAC and **(B)** DBNPA. Controls were performed with only live cells (C^−^) and dead cells, killed by autoclave (C^+^). The means ± SD of three independent experiments with triplicates are presented (RFU, relative fluorescence units).

The results obtained for resazurin assays (in RFU units) are presented in Figure 4.

Figure 4A, shows that BAC has a significant effect (*p* < 0.0001) on the reduction of cells’ metabolic activity as compared with the negative control (C^−^). More specifically, a 60–80% decrease in fluorescence intensity can be observed between BAC-exposed cells (for all tested concentrations) and the live ones from C^−^. Although the impact on cells’ metabolic activity is noticeable for concentrations below the MBC (100 mg/L), 160 mg/L was the only concentration presenting similar values to the autoclaved cells (C^+^; *p* > 0.05). These results suggest that the MBC concentration is not enough to decrease the cells metabolic activity to the levels observed in autoclaved cells.

Similarly, cells exposed to increasing DBNPA concentrations (Figure 4B) significantly decreased (*p* < 0.0001) the fluorescence intensity when comparing to C^−^. These results indicate that DBNPA affected the metabolic activity of cells (reductions between 58 and 78%), although without causing membrane damage. Also, the differences between C^+^ and the DBNPA concentrations tested were statistically significant, except for 35 mg/L (*p* > 0.05). Again, only the concentration above the MBC seems to be able to decrease the cells’ metabolic activity to levels comparable with the ones observed for the autoclaved (dead) cells.

#### Effect of Biocides on Cell Energy

**Figure 5:**
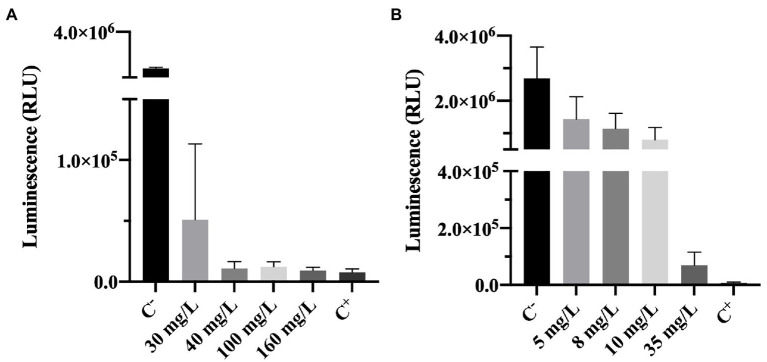
Luminescence of *Pseudomonas fluorescens* cells after being exposed for 30 min to different concentrations of **(A)** BAC and **(B)** DBNPA. Controls were performed with only live cells (C^−^) and dead cells, killed by autoclave (C^+^). The means ± SD of three independent experiments with triplicates are presented (RLU, relative light units).

Adenosine triphosphate bioluminescence assay is a powerful tool to assess cell viability (Lomakina et al., 2015). ATP is a key carrier of energy in cells, it is essential for cell respiration and its synthesis is immediately stopped after cell death (Lomakina et al., 2015). The ATP kit has a reagent that lyses cells. The principle of the method is that when cells are lysed ATP is released and the oxidation of luciferin (an organic substrate) by luciferase (an enzyme) uses ATP from viable cells to produce light. So, a higher luminescence (RLU) signal can be correlated to the presence of higher levels of ATP and, therefore, a higher number of viable cells (Lomakina et al., 2015; Kumar and Ghosh, 2019). As described in the methodology section, cells are lysed upon contact with one of the reagents present in the ATP test kit, enabling the detection of ATP. It is important to highlight that the ATP measured in this work (results shown in Figure 5) is the intracellular ATP (ATP present inside living cells), since upon biocide exposure, the samples were centrifuged and the supernatant discarded.

The luminescence results presented in Figure 5A show that increasing BAC significantly decreased the measured luminescence when compared to the positive control (*p* < 0.0001), suggesting a decrease on the ATP levels. Although from Figure 1A it was observed that 40 mg/L of BAC was far below the MBC (which was found to be 100 mg/L), the differences between 40, 100, 160 mg/L and C^−^ were not statistically significant (*p* > 0.05). These results seem to indicate that BAC concentrations equal to or above 40 mg/L decrease the ATP to levels similar to the ones from autoclaved dead cells (C^+^). In the work of Aragones et al. (2012), when *P. aeruginosa* was tested with 100 mg/L of BAC for 5 min of contact time, a 0.3 log_10_-reduction of luminescence was observed. Although this value is low compared to the 1.9 log_10_-reduction obtained in the present study, the contact time used was also lower and the microorganism (a Gram-negative) was not from the same species.

The comparison between Figure 5A (BAC) and Figure 5B (DBNPA) seems to indicate a smoother decreasing tendency between DBNPA concentration and luminescence decrease (Figure 5B) as compared to BAC, which can be related to the lytic action of BAC that leads to the release of intracellular contents (including ATP). Although luminescence decreases with increasing DBNPA concentrations (Figure 5B), there is a statistically difference (*p* < 0.0001) among all the tested concentrations and both controls (C^+^ and C^−^). The only exception is observed between the 35 mg/L and C^+^, which did not present statistically significant differences (*p* > 0.05). These results suggest that a 35 mg/L concentration of DBNPA is needed to decrease ATP to levels similar to the ones observed in cells submitted to an autoclaving process (C^+^).

#### Effect of Biocides on Cell Structure and Morphology

**Figure 6:**
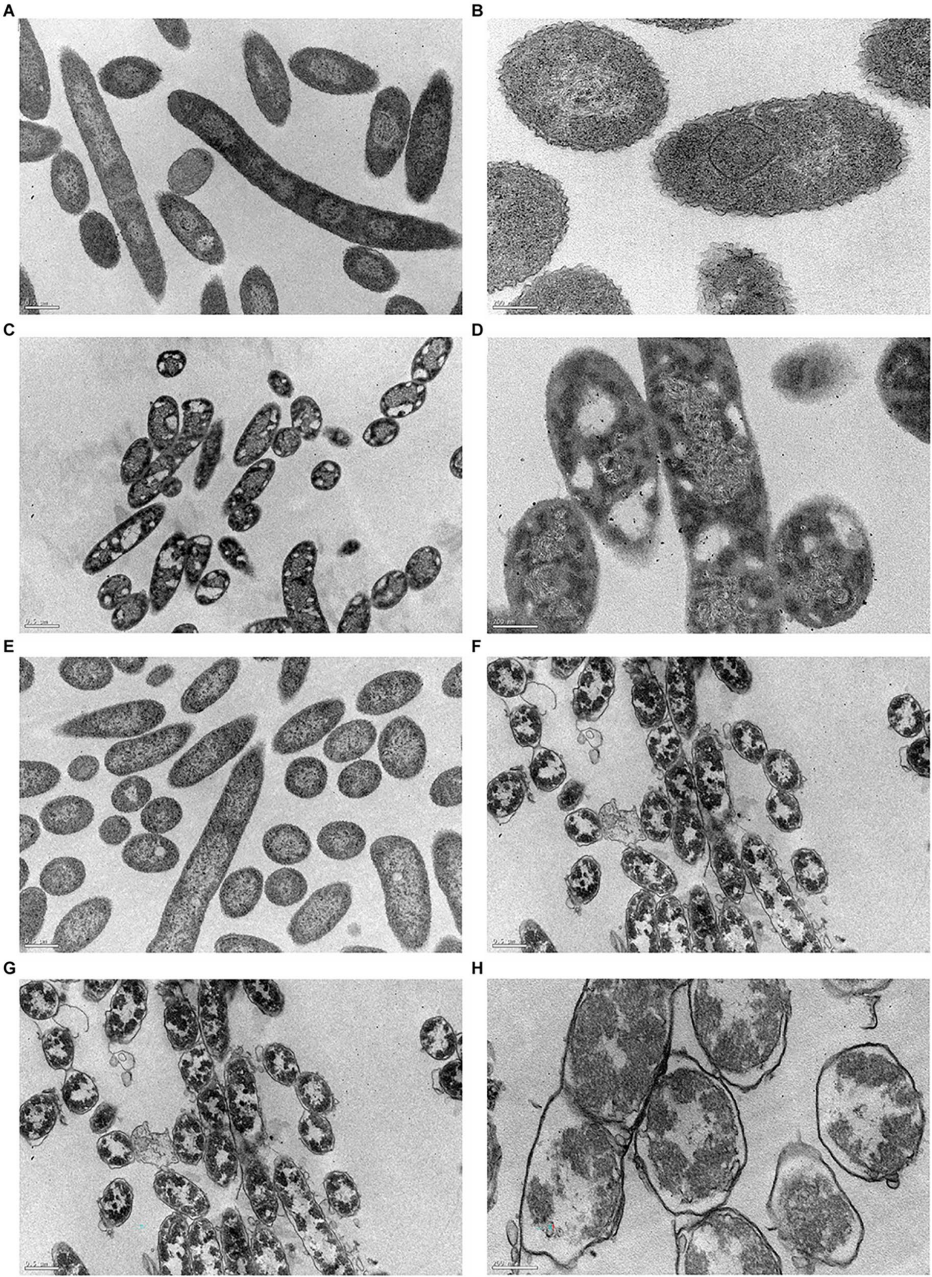
Transmission electron micrographs of *Pseudomonas fluorescens* cells: **(A,B)** untreated-C^−^; **(C,D)** exposed for 30 min to BAC minimum bactericidal concentration (MBC; 100 mg/L); **(E,F)** exposed to DBNPA MBC (10 mg/L); and **(G,H)** after an autoclaving process-C^+^. Images **(A,C,E,G)** are under a lower magnification (25,000×), while images **(B,D,F,H)** are at a higher magnification (80,000×).

In this work, TEM was used to accurately visualize biocide impact on bacterial cell envelope. For this study, we examined both controls (C^+^ and C^−^) and the cells previously exposed to BAC and DBNPA at the MBCs (100 and 10 mg/L, respectively; Figure 6).

Figures 6A,B revealed that untreated bacteria (C^−^) have a dense cytosol and a well-defined and intact bacterial cell envelope.

From Figures 6C,D is possible to notice the appearance of many vacuoles (islands of cytoplasm) that are not present in the C^−^. In a recent study, Ziklo et al. (2021) observed the same islands of cytoplasm on *P. aeruginosa* cells after dodecyl-dimethyl-ammonium chloride (a QAC compound) treatment. Also, BAC exposure led to a loss in intracellular content, with genetic material spread across the cytoplasm. Although there is no clear evidence of membrane disruption, the leaflets of bacterial cell envelope cannot be distinguished. The same trend has already been observed for colistin (Nguyen et al., 2021) and Polymyxin B (Voget et al., 2015), two antimicrobials which also target the outer membrane.

The exposure of cells to DBNPA (Figures 6E,F) appeared to have no effect on the bacterial cell envelope, but resulted in the loss of cellular components, which can be observed by the appearance of translucent zones.

Autoclaved cells (C^+^) Figures 6G,H-exhibited a high decrease in intracellular electron density. Besides, a lot of vesicles can be observed. Although the different layers of membrane structure cannot be distinguished, a massive cell wall damage was observed.

In short, all treatments induced structural and morphological changes to *P. fluorescens* and TEM images are in agreement with the results from the preceding analysis.

### Mechanistic Insights Into the Antimicrobial Action of BAC and DBNPA

The present work compares the antimicrobial effects of BAC and DBNPA and addresses the question “how far are injured cells from their dead state?”. This discussion is held for all the evaluated parameters (culturability, membrane integrity, metabolic activity, ATP, and TEM). The findings discussed so far, are summarized in Table 1.

**Table 1:** Summary of results obtained using different techniques to study the mechanisms of action of BAC and DBNPA.

Functional interpretation	Method	Findings	
		BAC Tested concentrations: 30, 40, 100 (MBC), and 160 mg/L	DBNPA Tested concentrations: 5, 8, 10 (MBC), and 35 mg/L
Culturability	Drop plate	Non-culturable cells (for concentrations equal or above the MBC)	Non-culturable cells (for concentrations equal or above the MBC)
Membrane integrity	PI+SYTO9 staining (BacLight)	Highly affected (even for concentrations lower than MBC)	Not affected (even for concentrations higher than MBC)
Metabolic activity	Resazurin	Highly reduced (only higher concentration are comparable to autoclaved dead cells)	Highly reduced (only higher concentration are comparable to autoclaved dead cells)
ATP	ATP Promega	Highly reduced (ATP decreased to levels similar to autoclaved dead cells – even for concentrations below MBC)	Reduced (only the concentration above MBC showed ATP levels comparable with autoclaved dead cells)
Structure and morphology	TEM	Vacuoles formation and loss of intracellular content	Intact membranes and loss of intracellular content

The most relevant observation that steps out from the results is the interaction of the two tested biocides with the cell’s membrane: while BAC clearly affects membrane integrity, DBNPA shows similar PI/SYTO9 uptake levels (no membrane damage) for all tested concentrations. However, both biocides, in higher or lower extent, interfered with the other evaluated parameters. These results will be discussed in the next paragraphs in light of the mechanisms of action proposed in the literature.

**Figure 7:**
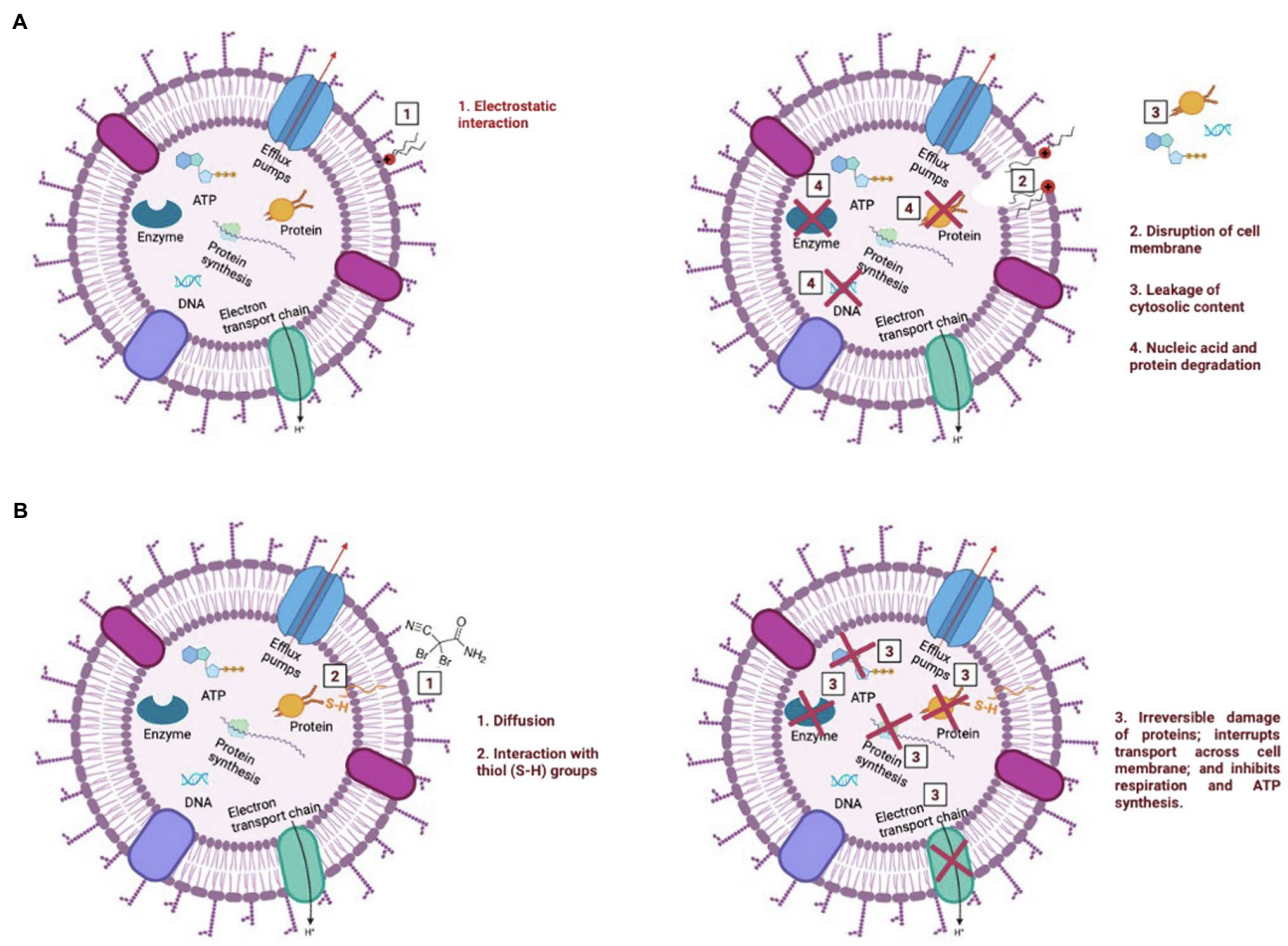
Illustration of the mechanism of action of BAC **(A)** and DBNPA **(B)**. These pictures were drawn based on what has been previously reported in the literature for BAC and DBNPA mechanisms of action (Salton, 1968; Maillard, 2002; McDonnell, 2007; Gerba, 2015; Campa et al., 2019).

Due to its cationic nature, BAC is known to bind electrostatically with the oppositely charged cell wall (Simões et al., 2005), followed by a sequence of events that include: (i) reaction with the cytoplasmatic membrane, leading to its disintegration; (ii) the intracellular constituents start to leak, followed by nucleic acids and proteins degradation; and (iii) autolytic enzymes lyse the cell wall (Salton, 1968; McDonnell, 2007; Gerba, 2015; see Figures 6A–D). As previously discussed, the interaction between BAC and the cell’s membrane is so strong that low BAC concentrations (below MBC) induce small membrane damages (pores), which are not enough to affect culturability, yet allow PI entrance, thereby providing a false indication of “compromised viability.” The induction of these pores on the cytoplasmatic membrane can lead to membrane disruption and leakage of intracellular contents, such as ATP (Nan et al., 2009; Suh et al., 2016). In fact, the exposure of bacterial cells to BAC led to a reduction of ATP content between 95 and 99%, as well as a decrease in the metabolic activity above 82%, for all tested concentrations. Although ATP decrease is often followed by viability loss (Suh et al., 2016; Yasir et al., 2019), only the highest tested concentration (above the MBC) showed ATP levels and a decreased metabolic activity comparable to the ones in autoclaved cells, even though bacteria lost their culturability after being exposed to BAC (in concentrations equal/above the MBC). The overall results reinforce that BAC is a lytic biocide (Chapman, 2003; Amjad, 2010; Bajpai, 2015; Figure 7A), since the membrane lost integrity, the cell lost the ability to grow in culture media (culturability), and also there was a decrease in both cell respiration and metabolic activity.

On the other hand, DBNPA’s mechanism of action relies on the interaction of bromine with sulfur-containing nucleophiles present inside the cells (e.g., cysteine or glutathione), leading to the inhibition of cell metabolism (Maillard, 2002; Campa et al., 2019; see Figure 7B). Such interaction between DBNPA and the components inside the cell (keeping the membrane intact) are corroborated in the present study by the membrane integrity and TEM results. When looking into culturability results, DBNPA exhibits a faster bactericidal activity than BAC, since it requires lower concentrations to achieve “total inactivation” (non-culturability) of bacterial cells for the same contact time. Furthermore, DBNPA-exposed cells showed an inhibition of cell metabolism, although in less extent than when BAC was applied. Nonetheless, similarly to BAC, DBNPA-exposed cells presented ATP and metabolic activity reduction levels consistent with the ones observed for autoclaved cells (C^+^) only for the highest tested concentration (35 mg/L)—the one above the MBC. This finding, common to both biocides, is related to the fact that the MBC concentration is not enough to decrease cell’s viability in all functional evaluated indicators (in the case of the present study: ATP and metabolic activity) and raises important issues regarding the interpretation/conclusions of when dead cells are effectively dead. If only the mechanisms of action were taken into account, bacterial cells exposed to 10 mg/L of DBNPA would be labelled as dead. However, the comparison of these injured cells with the autoclaved cells revealed that in fact the first ones were not really dead.

The basis of this study (as most reported in literature) is MBC’s determination—the lowest concentration able to decrease culturability to zero. Instead, if looking at ATP and resazurin results, other conclusions regarding the concentration to be used would be drawn. For example, relying on ATP and resazurin the indicative concentrations to apply would be: 160 mg/L (MBC from this study: 100 mg/L) for BAC and 35 mg/L (MBC from this study: 10 mg/L) for DBNPA. Thus, the answer to the former question “how far are injured cells from its dead state?”, follows the current understanding that “dead state” depends on the methodology used to evaluate it (Cangelosi and Meschke, 2014; Emerson et al., 2017). But, in the case of the present study, the inclusion of a positive control (autoclaved cells), allows us to add that the “dead state” seems to only occur for concentrations above the MBC, both for BAC and DBNPA. It is important to note that time is also a key factor when discussing antimicrobial performance of biocides, which in the case of the present study was kept constant—30 min. If higher contact times were considered, the findings herein discussed could be different.

#### The Issue of Viable but Non-culturable Cells

Over the past two decades, many researchers have been discussing that cells can reach the viable but non-culturable (VBNC) state as a mechanism of defense and self-preservation (Keep et al., 2006; Alam et al., 2007; Li et al., 2014; Emerson et al., 2017; Fleischmann et al., 2021; Kirschner et al., 2021). Cells enter the VBNC state when submitted to harsh stress conditions (Highmore et al., 2018; exposure to biocides/antibiotics, temperature shifts, lack of nutrients). The more usual characterization of cells that enter the VBNC state is: (i) they retain their membrane integrity; (ii) they are still metabolically active (reduced levels of metabolic activity); (iii) they are able to produce proteins; but (iv) they are not able to grow on solid media (Oliver, 2010; Ayrapetyan et al., 2014, 2018; Fleischmann et al., 2021; Kirschner et al., 2021). Also, VBNC cells are more chemically and physiologically resistant than culturable cells (Signoretto et al., 2000; Li et al., 2014), making them a major threat in many areas, including public health. If somehow the environmental conditions are restored, these cells can become culturable again. Hence, the detection of VBNC cells is important to prevent resuscitation, possible infections and adjust the biocide/antimicrobial dosing.

For illustration purposes, it could be considered that viability (for VBNC characterization) would be determined through PI/SYTO9 uptake results. Taking into consideration the mechanisms of action of the two biocides, this approach would clearly “penalize”/overestimate DBNPA in terms of VBNC calculations (Fleischmann et al., 2021), since this biocide does not interfere with the cell’s membrane, during the contact time considered. On the other hand, VBNC cells resulting from BAC exposure could be underestimated. This issue is particularly relevant when assessing VBNC’s in real-life systems, where biocides with different mechanisms of action are combinedly applied. Thus, the use of the most appropriate viability marker should be further discussed when determining VBNCs. Nonetheless, Kirschner et al. (2021) also pointed out that care must be taken when quantifying VBNC cells, especially when such quantification is based on a single viability parameter analysis. Alternatively, a double staining procedure with 4,6-di-amidino-2-phenylindole (DAPI) and tetrazolium salt 5-cyano-2,3-ditolyltetrazolium chloride (CTC) could be used to discriminate VBNC cells (Besnard et al., 2000; Fleischmann et al., 2021). Unlike PI/SYTO9, CTC does not assess membrane integrity, rather it is reduced through the electron transport chain by actively respiring bacteria (Wideman et al., 2021).

## Conclusion

The mechanisms of action of BAC and DBNPA were discussed in the present study. BAC was confirmed to be a lytic biocide, since it disrupts the cytoplasmatic membrane and releases the intracellular cell components. Also, DBNPA, as a moderate electrophilic biocide, did not interact with the membrane, but it interfered with key components of cell’s metabolism. The analysis of the results obtained by the different methods used (CFUs counting, ATP, resazurin, membrane integrity, and TEM) were in accordance to what has been previously proposed in the literature.

Furthermore, the current paper highlights an on-going discussion in the academic community regarding the need to combine different methodologies to assess cell’s viability and to screen different biocidal concentrations (both below and above MBC). Furthermore, the discussion of the antimicrobial efficacy of biocides should not only consider the outputs of viability assays, but also bring into the equation the mechanism of action. Finally, the introduction of a positive control by autoclaving the cells can help to dig into the question “how far are injured cells from its dead state?”

## Data Availability Statement

The raw data supporting the conclusions of this article will be made available by the authors, without undue reservation.

## Author Contributions

LM contributed to the funding acquisition. LM, AP, and AB conceptualized the study. AB performed the laboratory experiments. AP and AB contributed to writing of the original draft. LM and AP contributed to the discussion of results, review, and editing process. All authors contributed to the article and approved the submitted version.

## Funding

This work was financially supported by: (i) LA/P/0045/2020 (ALiCE), UIDB/00511/2020 and UIDP/00511/2020 (LEPABE), funded by national funds through FCT/MCTES (PIDDAC); (ii) Project pBio4.0—Preventing Biofouling in Membrane Systems, with the reference POCI-01-0247-FEDER-033298, co-funded by the European Regional Development Fund (ERDF), through the Operational Programme for Competitiveness and Internationalization (COMPETE 2020), under the PORTUGAL 2020 Partnership Agreement; and (iii) Project PRESAGE, with the reference Aquatic/0007/2020 and ID 375 of JPI-ERANET Aquatic Pollutants, co-financed by ERA-NET Cofund Aquatic Pollutants through the FCT. AB acknowledges the award of a Ph.D. grant from FCT (SFRH/BD/146028/2019).

## Conflict of Interest

The authors declare that the research was conducted in the absence of any commercial or financial relationships that could be construed as a potential conflict of interest.

## Publisher’s Note

All claims expressed in this article are solely those of the authors and do not necessarily represent those of their affiliated organizations, or those of the publisher, the editors and the reviewers. Any product that may be evaluated in this article, or claim that may be made by its manufacturer, is not guaranteed or endorsed by the publisher.
